# Radiological Response to Capecitabine and Temozolomide in an Elderly Patient with Metastatic Leiomyosarcoma: A Case Report

**DOI:** 10.3390/jcm15176668

**Published:** 2026-08-28

**Authors:** Suayib Yalcin, Can Kilinc, Taha Koray Sahin, Omer Dizdar

**Affiliations:** Department of Medical Oncology, Hacettepe University, Ankara 06800, Turkey; cank60@gmail.com (C.K.); takorsah@gmail.com (T.K.S.); dromerdizdar@gmail.com (O.D.)

**Keywords:** leiomyosarcoma, soft-tissue sarcoma, capecitabine, temozolomide, second-line chemotherapy, metastatic, elderly, case report

## Abstract

Metastatic leiomyosarcoma (LMS) is characterized by limited treatment options beyond first-line therapy, and cross-resistance among cytotoxic agents is often assumed to exist. However, differential sensitivity to distinct chemotherapy mechanisms may exist. We report the case of an 81-year-old woman diagnosed with metastatic LMS of unknown primary origin presenting with extensive hepatic and pulmonary involvement. Following eight cycles of first-line gemcitabine plus docetaxel, the patient achieved stable disease without objective tumor shrinkage. Given this limited response, a significant radiological response to subsequent therapy was not anticipated. Second-line oral capecitabine and temozolomide (CAPTEM) was initiated, resulting in a marked radiologic regression at four months, with reductions in hepatic metastases and pulmonary nodules and no new lesions. Treatment was well tolerated, with no grade 3–4 toxicities. This case demonstrates a differential response to gemcitabine-based therapy and sensitivity to alkylating agents in LMS, supporting a more individualized, mechanism-driven treatment sequencing approach.

## 1. Introduction

Leiomyosarcoma (LMS) is a malignant tumor arising from smooth muscle cells and accounts for approximately 10–20% of all soft-tissue sarcomas (STSs) [1]. It most commonly originates from the uterus, retroperitoneum, and vascular structures, though cases with no identifiable primary site have been reported [2,3]. Metastatic LMS is associated with a median overall survival of approximately 12–18 months, and treatment options in the second-line setting remain limited [4].

The treatment landscape for metastatic LMS has evolved in recent years. The phase III LMS04 trial demonstrated that induction therapy with doxorubicin plus trabectedin followed by trabectedin maintenance significantly improved both progression-free survival (12 vs. 6 months; HR, 0.37) and overall survival (33 vs. 24 months; HR, 0.65) compared with doxorubicin alone, establishing a new therapeutic benchmark for eligible patients [5]. Despite these advances, metastatic LMS remains an incurable disease for the majority of patients, and anthracycline-based regimens may not be suitable for elderly individuals because of comorbidities, limited physiological reserves, and concerns regarding treatment-related toxicity. Historically, ifosfamide-based combination regimens have also demonstrated meaningful activity in advanced soft-tissue sarcomas, with objective response rates ranging from 37.5% to 41.6% in phase II studies evaluating doxorubicin–ifosfamide and ifosfamide–etoposide combinations, respectively, highlighting the chemosensitivity of selected sarcoma subtypes to mechanism-specific cytotoxic approaches [6,7].

Beyond the first-line setting, there is no universally accepted standard of care. Several agents, including trabectedin, pazopanib, and dacarbazine-based regimens, have demonstrated modest efficacy [8,9]. Among these, temozolomide has shown consistent activity in LMS across multiple studies, raising the possibility that this histologic subtype may possess a distinct biological susceptibility to alkylating agents [10,11]. The capecitabine–temozolomide (CAPTEM) regimen was originally developed for neuroendocrine tumors based on preclinical evidence of synergy between capecitabine and temozolomide [12]. The proposed mechanism involves depletion of the DNA repair enzyme O^6^-methylguanine-DNA methyltransferase (MGMT), thereby enhancing the cytotoxic effects of temozolomide-induced DNA alkylation [13]. Whether a similar synergistic interaction exists in LMS remains uncertain. Nevertheless, the documented single-agent activity of temozolomide in LMS provides a biologically plausible rationale for exploring CAPTEM in this disease.

Here, we report the case of an 81-year-old woman with metastatic LMS of unknown primary origin who achieved significant radiologic tumor regression with second-line CAPTEM therapy, highlighting the potential for mechanism-specific chemosensitivity in this biologically heterogeneous disease.

## 2. Case Report

### 2.1. Initial Presentation and Diagnosis

An 81-year-old woman with a medical history of type 1 diabetes mellitus and chronic pain presented in February 2025 with abdominal complaints. She had no other major comorbidities and no history of previous malignancies. Her previous surgical history was unremarkable, and she was receiving insulin therapy for diabetes mellitus and symptomatic treatment for chronic pain. She had no known family history of malignancy. She was functionally independent in daily activities and had an Eastern Cooperative Oncology Group (ECOG) performance status of 1 at presentation. No additional clinically significant findings were identified on physical examination. Contrast-enhanced CT of the abdomen revealed multiple hypodense solid lesions throughout both hepatic lobes (>10 lesions, with the largest measuring 42 × 41 mm in Segment 7), highly indicative of metastatic disease. The presence of an exophytic left-kidney lesion measuring 19 × 19 mm with a fat density of −45 HU suggested angiomyolipoma. No pathologically enlarged lymph nodes were seen.

In March 2025, 18F-FDG PET/CT showed intensely FDG-avid hepatic lesions (SUVmax 13 in the left lobe), a right upper-lobe pulmonary nodule (1.2 cm, SUVmax 4.64), a sclerotic sacral lesion (SUVmax 3.86), mild FDG uptake in the left proximal humerus (SUVmax 4.94), and a subcutaneous nodule in the left mid-thigh (31 × 18 mm, SUVmax 7.94). Despite this widespread metastatic pattern, no primary tumor was seen.

In April 2025, a tru-cut biopsy of hepatic Segment 4 was performed (four cores, with the largest measuring 1.8 cm). Histopathological examination revealed a spindle cell tumor arranged in fascicles with generally low-grade nuclear atypia; occasional bizarre cells were noted. Mitoses were rare, and no coagulative necrosis was identified. Central hyalinization was present. Immunohistochemistry demonstrated positivity for smooth-muscle actin (SMA) and desmin, with negativity for S-100, DOG-1, and CD117. The Ki-67 proliferation index value was low (<10%), with focal areas approaching 10% on repeat staining. The patient’s morphological and immunohistochemical profiles were consistent with metastatic LMS. Given the diagnosis of LMS, potential uterine and vascular primary sites were specifically considered. Available pelvic imaging demonstrated no uterine masses or other radiological findings suggestive of a uterine primary origin. A diagnosis of metastatic LMS of unknown primary origin was established.

### 2.2. First-Line Treatment

Following multidisciplinary-tumor-board evaluation, the patient was started on first-line chemotherapy with docetaxel (75 mg/m^2^, day 1) and gemcitabine (900 mg/m^2^, days 1 and 8) every 21 days starting in May 2025. Baseline CT (May 2025) confirmed the presence of multiple bilateral hepatic metastases, with the largest measuring 4.5 cm; a right major fissure nodule measuring 17 × 12 mm; and bilateral parenchymal pulmonary nodules. The left adrenal region contained a 3.3 cm lesion indeterminate between a gastric diverticulum and an adrenal mass.

Interim CT at cycle 4 (July 2025) showed hepatic stable disease and slight regression of pulmonary nodules. Subsequent evaluations in October and December 2025 confirmed the presence of stable disease. After the completion of eight cycles (through November 2025), CT re-staging (December 2025) demonstrated stable hepatic metastases (largest 4.5 cm) and stable pulmonary nodules (with a right major fissure nodule measuring 8 × 7 mm). The best response was stable disease (SD) per RECIST 1.1. The treatment was generally well-tolerated; the patient’s comorbid diabetes required ongoing management throughout therapy.

### 2.3. Second-Line Treatment: CAPTEM

Following completion of eight cycles of docetaxel plus gemcitabine in November 2025, restaging CT performed in December 2025 demonstrated stable disease without an objective response. Following this assessment, and considering the patient’s advanced age and comorbidities, the multidisciplinary tumor board recommended second-line therapy with oral capecitabine–temozolomide (CAPTEM), which was subsequently initiated. The regimen consisted of administering 750 mg/m^2^ of capecitabine orally twice daily on days 1–14 plus 200 mg/m^2^ of temozolomide orally once daily on days 10–14, repeated every 28 days.

**Figure 1:**
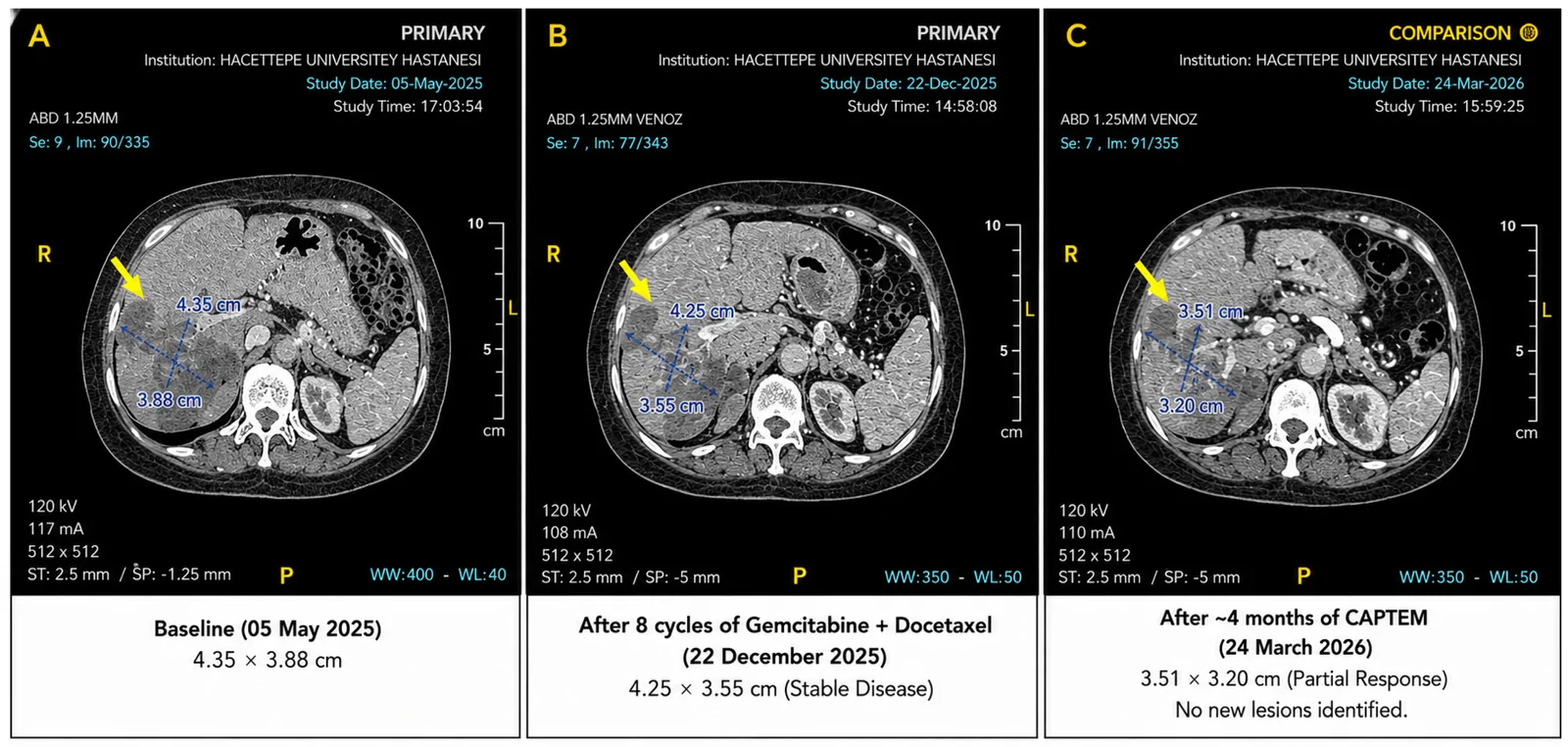
Serial contrast-enhanced CT images obtained in the venous phase demonstrating the treatment response of the same target hepatic lesion (arrows) at comparable anatomical levels. (**A**) Baseline CT (5 May 2025) image showing the target hepatic metastasis measuring 4.35 × 3.88 cm. (**B**) CT image obtained after eight cycles of gemcitabine plus docetaxel (22 December 2025), showing an essentially unchanged lesion measuring 4.25 × 3.55 cm, consistent with stable disease. (**C**) CT image obtained after approximately four months of CAPTEM therapy (24 March 2026), demonstrating a reduction in the target lesion to 3.51 × 3.20 cm. No new lesions were identified.

A response assessment CT scan performed at Hacettepe University Hospital in March 2026 (approximately 4 months after CAPTEM initiation) demonstrated radiological regression of hepatic metastases. The representative target hepatic lesion illustrated in Figure 1 had decreased from 4.35 cm at baseline to 3.51 cm at its longest diameter, and the majority of hepatic lesions showed interval size reduction. No new hepatic or extrahepatic lesions were identified. Pulmonary reassessment showed that the right major fissure nodule had decreased from 8 × 7 mm to 6 × 3 mm, with bilateral pulmonary nodules remaining stable. The adrenal/gastric region lesion was identified as a gastric fundal diverticulum on follow-up imaging, with no adrenal mass confirmed. These findings were consistent with a favorable radiologic response. The tumor board recommended continuation of CAPTEM therapy.

**Figure 2:**
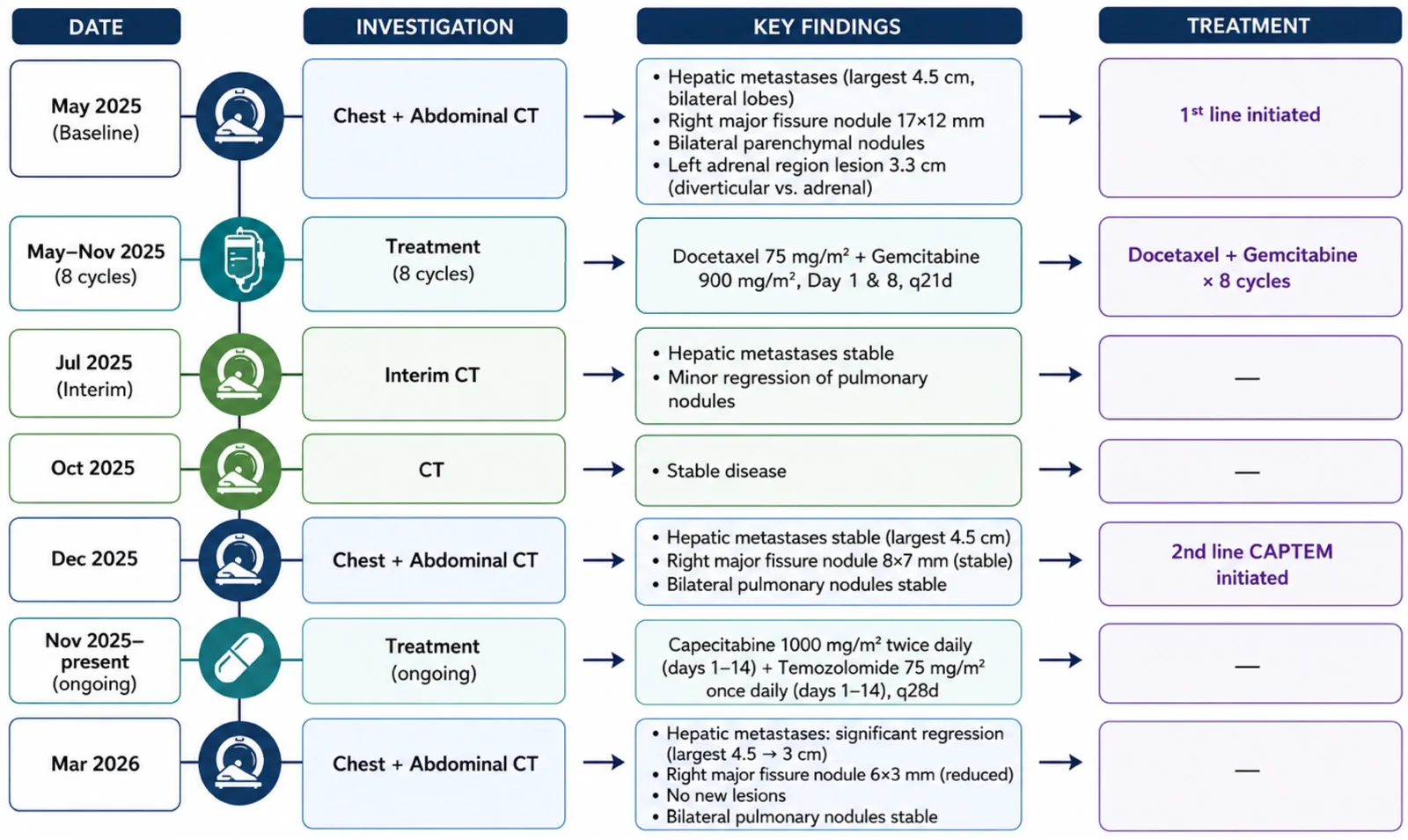
Clinical and radiological timeline of the disease course, treatment sequence, and radiological assessments.

The CAPTEM regimen was tolerated without significant grade 3–4 toxicities. The patient maintained her performance status throughout treatment and reported no dose-limiting adverse events requiring treatment discontinuation or dose reduction. A comprehensive overview of the patient’s clinical course and treatment milestones is presented in Figure 2, while representative serial radiological responses are illustrated in Figure 2. This case report was prepared in accordance with the CARE (CAse REport) Guidelines (Table S1).

## 3. Discussion

The present case demonstrates an unusual pattern of therapeutic response in metastatic leiomyosarcoma characterized by prolonged disease stabilization with gemcitabine–docetaxel followed by a substantial radiologic response to second-line CAPTEM. Although this sequential pattern of response is clinically noteworthy, a single case cannot establish differential or mechanism-specific chemosensitivity. Rather, this observation should be considered hypothesis-generating and interpreted in the context of the limited clinical evidence regarding applying CAPTEM to LMS.

The therapeutic landscape of metastatic LMS remains challenging. Although the recent LMS04 trial established doxorubicin plus trabectedin followed by trabectedin maintenance as a preferred first-line strategy for eligible patients, most individuals with advanced LMS ultimately experience disease progression and require subsequent therapies [5]. Among elderly patients, treatment selection is further complicated by comorbidities, reduced physiological reserves, and concerns regarding treatment-related toxicity. Consequently, identifying effective and well-tolerated therapeutic alternatives remains an important unmet clinical need.

Gemcitabine-based regimens, particularly in combination with docetaxel, are widely used in LMS treatment and have demonstrated meaningful clinical activity [14,15]. However, treatment responses are heterogeneous, and a substantial proportion of patients achieve only disease stabilization without objective tumor regression, as observed in our case. The subsequent radiological response to CAPTEM therefore demonstrates a differential response to two cytotoxic regimens with distinct mechanisms of action.

The observed differential response may be explained by the fundamentally distinct mechanisms of action of the two regimens. While gemcitabine exerts its cytotoxic effect through inhibition of DNA synthesis in proliferating cells, temozolomide induces DNA damage via O6-methylguanine adduct formation, triggering mismatch-repair-mediated apoptosis in a manner that is not strictly dependent on active cell cycling [16]. This distinction may be particularly important in tumors with low proliferative activity. We hypothesize that the very low Ki-67 proliferation index value observed for our patient may have accounted for the limited susceptibility to S phase-dependent agents while maintaining vulnerability to alkylation-induced DNA damage. Prospective validation of this hypothesis is required.

The biological rationale for CAPTEM has largely been derived from studies on neuroendocrine neoplasms, where capecitabine is thought to potentiate the activity of temozolomide through depletion of the DNA repair enzyme MGMT [17]. Whether a similar synergistic effect exists in leiomyosarcoma remains unclear. Nonetheless, several retrospective analyses and small clinical series have consistently reported clinically meaningful activity of temozolomide in LMS, raising the possibility that this histologic subtype may harbor a distinct susceptibility to alkylating agents [10,11]. The radiological response observed in our patient provides additional clinical support for this concept.

Beyond gemcitabine-based regimens, current NCCN guidance lists pazopanib, trabectedin (category 1 in LMS), eribulin, dacarbazine, and gemcitabine/dacarbazine as accepted second-line and subsequent options, each providing generally modest activity [18,19]. Importantly, the available evidence for temozolomide monotherapy in regard to LMS is derived primarily from small phase II studies and retrospective series, whereas direct evidence supporting the capecitabine–temozolomide combination specifically in regard to LMS remains very limited [20,21]. The rationale for CAPTEM is largely extrapolated from experience relating to neuroendocrine neoplasms. Accordingly, the present observation should be regarded as hypothesis-generating regarding potential alkylator sensitivity rather than as evidence validating CAPTEM as an established treatment regimen for LMS.

From a clinical standpoint, this case highlights the importance of maintaining therapeutic flexibility in metastatic LMS, particularly in elderly patients, where treatment tolerability often plays a central role in decision-making. The oral administration and favorable safety profile of CAPTEM make it an attractive option in this setting. More importantly, our observation suggests that a limited response to one class of cytotoxic agents should not necessarily discourage the use of subsequent therapies with distinct mechanisms of action, as clinically meaningful responses may still be achieved.

The limitations of this report include its single-patient nature, the absence of comprehensive molecular profiling, and the lack of MGMT expression or promoter methylation analyses. Nevertheless, the magnitude and consistency of the radiologic response across multiple metastatic sites provide a compelling signal of biological activity. Further translational studies and prospective clinical investigations are warranted to better define the role of alkylating agent-based strategies, including CAPTEM, in selected patients with metastatic LMS.

## 4. Conclusions

This case demonstrates that clinically meaningful responses to alkylating agent–based therapy may occur in metastatic LMS despite limited response to prior gemcitabine-based chemotherapy. The remarkable radiologic regression observed with CAPTEM in an elderly patient with extensive metastatic disease suggests that chemosensitivity in LMS may be determined more by tumor biology and mechanism-specific vulnerabilities than by generalized cytotoxic responsiveness. In addition to its favorable tolerability profile, CAPTEM may represent a valuable therapeutic option for selected patients who are unsuitable for intensive treatment approaches. Although conclusions cannot be drawn from a single case, this observation highlights the need for further translational and clinical studies investigating predictive biomarkers and mechanism-based treatment sequencing strategies in LMS.

## Data Availability

No new data were created or analyzed in this study. Data sharing is not applicable to this article.

## Supplementary Materials

The following supporting information can be downloaded at: https://www.mdpi.com/article/10.3390/jcm15176668/s1, Table S1: CARE Checklist of Information to Include When Writing a Case Report.

## Abbreviations

The following abbreviations are used in this manuscript:

CAPTEM	capecitabine plus temozolomide
CR	complete response
CT	computed tomography
FDG	fluorodeoxyglucose
HR	hazard ratio
LMS	leiomyosarcoma
MGMT	O6-methylguanine-DNA methyltransferase
MRI	magnetic resonance imaging
NCCN	National Comprehensive Cancer Network
ORR	objective response rate
PET/CT	positron emission tomography/computed tomography
PR	partial response
RECIST	Response Evaluation Criteria in Solid Tumors
SD	stable disease
STS	soft-tissue sarcoma
SUVmax	maximum standardized uptake value
