# Supplementary material for: Radiological Response to Capecitabine and Temozolomide in an Elderly Patient with Metastatic Leiomyosarcoma: A Case Report
Source: J Clin Med. 2026 Aug 28;15(17):6668. doi: 10.3390/jcm15176668 (PMC13566316; doi:10.3390/jcm15176668)
Supplement: Supplementary file 1 [file jcm-15-06668-s001.zip › jcm-4449009-supplementary.pdf]

Table S1. CARE Checklist of Information to Include When Writing a Case Report

Manuscript: Remarkable Radiological Response to Capecitabine and Temozolomide in an Elderly Patient with Metastatic Leiomyosarcoma: A Case Report

Topic	Item	Checklist item description	Reported on Line
Title	1	The diagnosis or intervention of primary focus followed by the words "case report".	Lines 2-4
Key Words	2	2 to 5 key words that identify diagnoses or interventions in this case report, including "case report".	Keywords section; revised
Abstract	3a	Introduction: What is unique about this case and what does it add to the scientific literature?	Lines 8-13
Abstract	3b	Main symptoms and/or important clinical findings.	Lines 9-12
Abstract	3c	The main diagnoses, therapeutic interventions, and outcomes.	Lines 9-20
Abstract	3d	Conclusion - What is the main "take-away" lesson(s) from this case?	Lines 20-24
Introduction	4	One or two paragraphs summarizing why this case is unique (may include references).	Lines 25-78
Patient Information	5a	De-identified patient-specific information.	Lines 80-82
Patient Information	5b	Primary concerns and symptoms of the patient.	Lines 80-82
Patient Information	5c	Medical, family, and psycho-social history including relevant genetic information.	Lines 80-82 (relevant medical history); family/psychosocial history not contributory
Patient Information	5d	Relevant past interventions with outcomes.	N/A - no relevant prior oncologic intervention
Clinical Findings	6	Describe significant physical examination and important clinical findings.	Clinical findings: Lines 80-101; no significant PE finding reported
Timeline	7	Historical and current information from this episode of care organized as a timeline.	Figure 1; Lines 132-134
Diagnostic Assessment	8a	Diagnostic testing (such as PE, laboratory testing, imaging, surveys).	Lines 81-101
Diagnostic Assessment	8b	Diagnostic challenges (such as access to testing, financial, or cultural).	N/A - no relevant diagnostic access/financial/cultural challenge
Diagnostic Assessment	8c	Diagnosis (including other diagnoses considered).	Lines 92-101
Diagnostic Assessment	8d	Prognosis (such as staging in oncology) where applicable.	Lines 81-101; metastatic disease described
Therapeutic Intervention	9a	Types of therapeutic intervention (such as pharmacologic, surgical, preventive, self-care).	Lines 102-124
Therapeutic Intervention	9b	Administration of therapeutic intervention (such as dosage, strength, duration).	Lines 102-124
Therapeutic Intervention	9c	Changes in therapeutic intervention (with rationale).	Lines 115-124
Follow-up and Outcomes	10a	Clinician- and patient-assessed outcomes (if available).	Lines 108-131
Follow-up and Outcomes	10b	Important follow-up diagnostic and other test results.	Lines 108-131; Figure 2, Lines 138-144
Follow-up and Outcomes	10c	Intervention adherence and tolerability (How was this assessed?).	Lines 125-131
Follow-up and Outcomes	10d	Adverse and unanticipated events.	Lines 125-131; no grade 3-4 toxicities reported

Topic	Item	Checklist item description	Reported on Line
Discussion	11a	A scientific discussion of the strengths AND limitations associated with this case report.	Lines 145-223
Discussion	11b	Discussion of the relevant medical literature with references.	Lines 145-214
Discussion	11c	The scientific rationale for any conclusions (including assessment of possible causes).	Lines 166-214
Discussion	11d	The primary "take-away" lessons of this case report (without references) in a one-paragraph conclusion.	Lines 215-223
Patient Perspective	12	The patient should share their perspective in one to two paragraphs on the treatment(s) they received.	Not available / not reported
Informed Consent	13	Did the patient give informed consent? Please provide if requested.	Lines 235-237; Yes

Note: The checklist is completed against the current/revised manuscript structure. The manuscript should explicitly state adherence to the CARE Guidelines. The patient perspective was not available and therefore is transparently marked as not reported rather than inferred.
